# Major Harry M. Hallock, M. D.

**Published:** 1913-09

**Authors:** 


Our August issue contained an obituary of Major Harry M. Hallock, M. D., formerly stationed at Fort Porter. After the notice was in type, we found a personal letter from him, inclosed in a report of the Government Reservation at Hot Springs, received some weeks previously but not opened. The tone of his letter was so cheerful that we are inclined to regard his shooting as accidental and not suicidal, as reported. His letter carried greetings to his many Buffalo friends.

